# Thermal expansion of Pd-based metallic glasses by *ab initio* methods and high energy X-ray diffraction

**DOI:** 10.1038/s41598-017-16117-7

**Published:** 2017-11-16

**Authors:** Simon Evertz, Denis Music, Volker Schnabel, Jozef Bednarcik, Jochen M. Schneider

**Affiliations:** 1 Materials Chemistry, RWTH Aachen University, Kopernikusstr. 10, 52074 Aachen, Germany; 2 Laboratory for Nanometallurgy, ETH Zürich, Vladimir-Prelog-Weg 5, 8093 Zurich, Switzerland; 3 Deutsches Elektronen-Synchrotron DESY, FS-PE Group, Notkestr. 85, 22607 Hamburg, Germany

## Abstract

Metallic glasses are promising structural materials due to their unique properties. For structural applications and processing the coefficient of thermal expansion is an important design parameter. Here we demonstrate that predictions of the coefficient of thermal expansion for metallic glasses by density functional theory based *ab initio* calculations are efficient both with respect to time and resources. The coefficient of thermal expansion is predicted by an *ab initio* based method utilising the Debye-Grüneisen model for a Pd-based metallic glass, which exhibits a pronounced medium range order. The predictions are critically appraised by *in situ* synchrotron X-ray diffraction and excellent agreement is observed. Through this combined theoretical and experimental research strategy, we show the feasibility to predict the coefficient of thermal expansion from the ground state structure of a metallic glass until the onset of structural changes. Thereby, we provide a method to efficiently probe a potentially vast number of metallic glass alloying combinations regarding thermal expansion.

## Introduction

Metallic glasses are promising materials for micromechanical structural applications due to the combination of high strength and elasticity, hardness and toughness^1–3^, leading to an outstanding property combination compared to other material classes^4^. In addition to these property combinations, the absence of microstructural features^2^ makes metallic glasses ideal for micromechanical applications. The low viscosity in the supercooled liquid region allows the near net-shape production of structural components by thermoplastic forming, while volume shrinkage during casting is one magnitude lower compared to crystalline materials due to the lack of a first order phase transition during solidification^5^. For processing by near net shape production methods and application in micromechanical devices the coefficient of thermal expansion (CTE) is of critical importance in terms of dimensional accuracy and thermal stresses^1–3,5^.

Pd-based metallic glasses exhibit promising property combinations such as high strength and toughness, leading to damage tolerant materials^6^. Schnabel *et al*. reported a metalloid-free Pd_57.0_Al_23.9_Y_7.7_Cu_11.4_ metallic glass with extensive plastic deformation before fracture and a high damage tolerance^4^. Following the electronic structure based design proposal of Schnabel *et al*. that a low fraction of hybridised bonds is a fingerprint of damage-tolerance^4^, Pd_62.1_Al_27.3_Y_4.2_Ni_6.4_ is predicted to exhibit damage tolerance. In addition to the damage tolerance, the knowledge of the CTE is crucial for potential micromechanical applications.

While knowledge based *ab initio* models to predict structure and mechanical, electrical and magnetic properties of metallic glasses are state of the art^7–10^, there are no reports on Debye-Grüneisen model to predict the CTEs of metallic glasses in literature. The CTEs of cubic^11–17^, tetragonal^16,17^, hexagonal and trigonal materials^17^ have been calculated by the Debye-Grüneisen model. To our knowledge, the Debye-Grüneisen model has not been applied to amorphous materials yet. Here it is demonstrated that the *ab initio* based prediction of CTEs is efficient with respect to time and resources. As a model system we have evaluated a Pd_54.7_Al_23.5_Y_7.8_Ni_11.3_ thin film metallic glass by the *ab initio* based Debye-Grüneisen model and validated the theoretical predictions by high energy X-ray diffraction experiments.

## Methods

In this work, *ab initio* molecular dynamic simulations were carried out using the density functional theory^18^ based openMX code^19,20^. Electronic potentials with the generalised gradient approximation were employed^21^. Basis functions were linear combinations of localised pseudoatomic orbitals^22^. The following basis functions were applied: Pd5.0-s2p1d1, Al6.0s1p2, Y6.5-s3p2d1 and Ni6.0-s2p2d2f1. The first symbol designates the chemical element followed by the cutoff radius. The last set of symbols defines the primitive orbitals. An N-point grid of 85 × 85 × 85 and a cutoff energy of 150 Ry has been used. For volume relaxation at 0 K the Vienna *Ab-initio* Simulation Package was utilised^23,24^. Thereby, the ultrasoft pseudopotentials were employed and the Brillouin zone was integrated on a 3 × 3 × 3 Monkhorst-Pack *k*-point grid^25^.

To model both short- and medium-ranged order, the structural model introduced by Hostert *et al*.^8^ was modified in terms of the size of the supercell and of relaxation time. Here, a larger supercell containing 389 atoms and 43 vacancies was employed. In order to obtain an amorphous structure, the supercell was heated to 4000 K for 4000 fs and quenched to 0 K with infinite cooling rate until the volume change between two subsequent cycles was <2%. As described previously by Hostert *et al*.^8^, the application of an infinite cooling rate is appropriate compared to cooling rates accessible in thin film synthesis. The bulk modulus B was obtained from the ground state by fitting the volume-energy data with the Birch-Munarghan equation of state^26^. Shear and Young’s modulus were calculated by means of volume conservative distortions^27^. In order to allow a medium range order to develop, relaxation of the atomic positions was allowed at 300, 400, 700 and 800 K for 4400 fs. Taking the scattering powers of the constituents into account, the pair distribution function (PDF) g(r) was calculated from the atomic positions^8^.

To estimate the CTE, a Debye-Grüneisen theory based method described by Söderlind *et al*.^11^ was used. Thereby the Helmholtz free energy (eq. (1)) was calculated for different temperatures.

1 $$F(r,T)={E}_{e}(r)-N{k}_{B}T[3{(\frac{T}{{\rm{\Theta }}})}^{3}{\int }_{0}^{\frac{{\rm{\Theta }}}{T}}\frac{{x}^{3}}{{e}^{x}-1}dx-3\,\mathrm{ln}(1-{e}^{-\frac{{\rm{\Theta }}}{T}})-\frac{9{\rm{\Theta }}}{8T}]$$

E_e_(r) is the temperature independent electron energy, N the number of atoms in the supercell, k_B_ Boltzmann’s constant and T the temperature. From the mechanical properties obtained in the *ab initio* calculations, namely bulk modulus B and Poisson’s ration ν, the Debye temperature Θ was calculated by eq. (2)

2 $${\rm{\Theta }}=\frac{h}{{k}_{B}}{(\frac{4\pi }{3})}^{-\frac{1}{6}}{[\frac{2}{3}{(\frac{2}{1-2\nu })}^{\frac{3}{2}}+\frac{1}{3}{(\frac{1}{1-\nu })}^{\frac{1}{3}}]}^{-\frac{1}{3}}{(\frac{3}{1+\nu })}^{\frac{1}{2}}{(\frac{rB}{M})}^{\frac{1}{2}}$$

h is the Planck constant and M the atomic mass. The dependence of the free energy on the Wigner-Seitz radius r was introduced via the change of Debye Θ temperature with r (eq. (3)), whereby γ is the Grüneisen parameter and r_0_ the equilibrium radius.

3 $${\rm{\Theta }}={{\rm{\Theta }}}_{0}{(\frac{{r}_{0}}{r})}^{3\gamma }$$

From the free energy curve calculated for several temperatures the equilibrium radii at each temperature were calculated. From the change of the equilibrium radius r_0_ with temperature T, the linear and volumetric thermal expansion coefficients α_lin_ and α_vol_, respectively, were obtained by eq. (4)

4 $${\alpha }_{lin}(T)=\frac{1}{3}{\alpha }_{vol}=\frac{1}{{r}_{0}(T)}\frac{d{r}_{0}(T)}{dT}$$

To measure the thermal expansion by synchrotron X-ray diffraction Pd_62.1_Al_27.3_Y_4.2_Ni_6.4_ thin films were deposited on rotating NaCl substrates by magnetron sputtering of elemental targets in DC mode in an ultra-high vacuum chamber (base pressure <51 mPa). Floating bias was utilised, the power densities on the Pd, Al, Y and Ni targets were 4.2, 4.7, 1.7 and 1.3 W/cm^2^, respectively. Ar was employed as working gas with a pressure of 0.4 Pa during sputtering. For the synchrotron measurements, the NaCl substrate was dissolved in water to obtain thin film particles, which were subsequently cleaned in isopropanol and acetone.

High-energy X-ray diffraction was conducted at beamline P02.1 of the PETRA III electron storage ring at DESY, Hamburg. X-rays with a wavelength of 0.20701 Å were used and data collected with a Perkin Elmer XRD1621 fast detector. The sample was positioned in a Linkam THMS 600 heating stage and heated up with a heating rate of 10 K/min up to 863 K, hold at this temperature for 300 s and cooled down to room temperature with 10 K/min. Diffraction patterns were taken continuously with a measurement and detector read-out time of 12 s. The diffraction patterns were integrated over this time interval, which leads to a temperature resolution of 2 K. We used the FIT2D software^28–30^ to process the diffraction patterns.

The thermal expansion of an amorphous substance is connected to the shift of the principal peak position Q in reciprocal space as described in eq. (5)^31^, where Q is the principal peak position, V the atomic volume and α_vol_ the volumetric CTE. T_0_ indicates the reference temperature, in this study room temperature.

5 $${\{\frac{Q({T}_{0})}{Q(T)}\}}^{3}=\frac{V(T)}{V({T}_{0})}=1+{\alpha }_{vol}(T-{T}_{0})$$

The principal peak was fitted by a Pseudo-Voigt function in order to obtain the quantitative peak positions. Throughout this work, all CTEs are given as volumetric CTEs.

To study the structural changes during heating, the structure factor S(Q) and the reduced PDF G(r) were obtained using the PDFgetX3 software^32^. From the structure factor S(Q) the reduced PDF G(r) was calculated by a Fourier transformation. g(r) and G(r) are connected by

6 $$g(r)=\frac{\rho (r)}{{\rho }_{0}}=\frac{G(r)}{4\pi {\rho }_{0}r}+1$$

## Results and Discussion

In this part, first the CTE of Pd_54.7_Al_23.5_Y_7.8_Ni_11.3_ metallic glass is predicted by the Debye-Grüneisen model and critically appraised by *in situ* X-ray diffraction. Thereafter, the calculated and experimentally observed structures are compared to validate the employed *ab initio* model. In the third part, a refined structural analysis is conducted to investigate the changes in the atomic structure observed in the experiments.

### Thermal expansion

**Figure 1:**
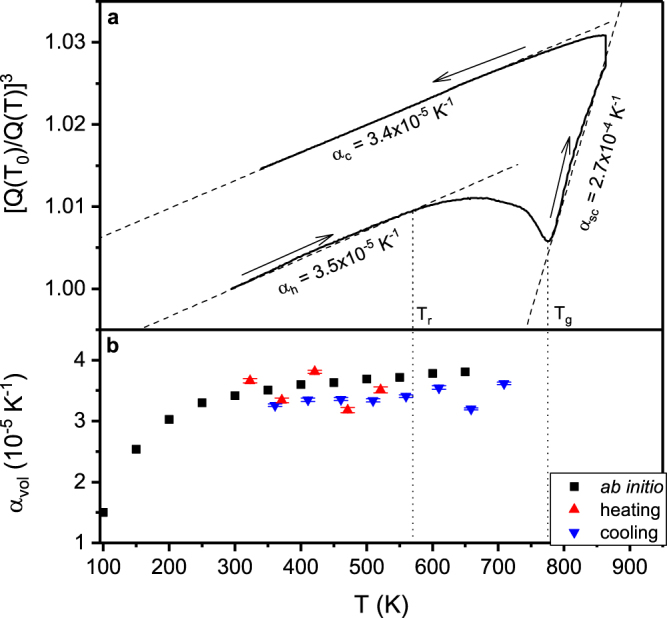
Thermal expansion of a Pd based metallic glass. (**a**) Ratio of the principle peak positions of the structure factor (Q(T_0_)/Q(T))^3^ of Pd_62.1_Al_27.3_Y_4.2_Ni_6.4_ as a function of temperature. The dashed lines are linear fits with the slopes as the volumetric coefficient of thermal expansion in the heating, cooling and supercooled region α_h_, α_c_ and α_sc_, respectively. Dotted lines mark the relaxation and glass transition temperature T_r_ and T_g_. Arrows indicate the direction of heating. (**b**) Coefficient of thermal expansion of Pd_54.7_Al_23.5_Y_7.8_Ni_11.3_ by *ab initio* based Debye-Grüneisen model. Debye temperature is 332 K. The red and blue triangles represent the experimental CTEs in the heating and cooling sections, respectively.

We evaluate the thermal expansion of Pd_62.1_Al_27.3_Y_4.2_Ni_6.4_ thin film metallic glasses by *in situ* X-ray diffraction and by *ab initio* methods utilising the Debye-Grüneisen model. Figure 1(a) shows the synchrotron X-ray diffraction results for the thermal expansion of Pd_62.1_Al_27.3_Y_4.2_Ni_6.4_ thin film powder. The ratio of the principal peak positions (Q(T_0_)/Q(T))^3^ of the structure factor S(Q) is depicted as a function of temperature since this ratio is directly related to volume changes in the glass^31,33,34^. Thus, the CTE is related to (Q(T_0_)/Q(T))^3^ below T_g_ provided that no structural change occurs^31^, as has been reported for several La-, Pd-, Sm-, Co- and Zr-based metallic glasses^31,33–40^. In the heating section, beginning at room temperature, the ratio (Q(T_0_)/Q(T))^3^ is increasing linearly. From 570 K onwards (this temperature is referred to as relaxation temperature T_r_), the CTE decreases and eventually turns negative at T_n_ = 670 K. The (Q(T_0_)/Q(T))^3^ curve reaches a minimum at 775 K. According to Bednarcik *et al*.^34^ the minimum in the (Q(T_0_)/Q(T))^3^ curve corresponds to the glass transition temperature T_g_. The continuing change in the (Q(T_0_)/Q(T))^3^ ratio due to homogenisation of the sample temperature at the maximum temperature of 863 K during a holding time of 5 min causes an infinite slope. In the cooling section, the (Q(T_0_)/Q(T))^3^ ratio decreases linearly. The linear regions of the heating and cooling sections are fitted in intervals of 50 K. The resulting CTEs are shown in Fig. 1(b). The average in CTE for the heating and cooling section are 3.5 ± 0.2 × 10^−5^ and 3.4 ± 0.1 × 10^−5^ K^−1^, respectively, which is consistent with CTE for other Pd-based metallic glasses^41^. In the supercooled region above T_g_ we measured a CTE of 2.7 × 10^−4^ K^−1^, which is 8 times larger than the CTEs in the glassy regions. This can be rationalized based on additional excitations of atomic oscillators at the glass transition^33,34,42^. In contrast to the thermal expansion while cooling, the thermal expansion while heating begins to deviate from a linear dependence at T_r_. Throughout the whole cooling region a linear dependency of (Q(T_0_)/Q(T))^3^ to the temperature is observed. The non-linear dependency of (Q(T_0_)/Q(T))^3^ on temperature while heating is caused by the annihilation of free volume in the structure^31,33,43^. The CTEs in the heating and cooling section are consistent within the error of the measurement. This holds because the difference in free volume between the heating and cooling curves does not influence the relative shift of the principle maximum of S(Q) in the relaxation free temperature region^33^. While heating the metallic glass, excess free volume is annihilated. Hence, with increasing temperature the thermal expansion is superimposed by free volume annihilation. Above T_r_ the influence of free volume annihilation on the macroscopic thermal expansion is larger than the thermal volume expansion. Eventually, this causes a negative CTE at 670 K.

Figure 1(b) depicts the calculated CTEs from the Debye-Grüneisen model based on the *ab initio* calculations in the ground state and the experimental CTEs as a function of temperature. The calculated CTEs increase between 100 and 300 K from 1.5 × 10^−5^ to 3.4 × 10^−5^ K^−1^. Above the Debye temperature Θ_D_ = 332 K, the CTE increases only slightly. Hence, we observe excellent agreement between the experimentally and theoretically obtained CTEs of the Pd_54.7_Al_23.5_Y_7.8_Ni_11.3_ thin film metallic glass, which is within the error of the measurement and the precision of the Debye temperature calculation. The Debye temperature depends on the temperature dependent equilibrium volume, elastic constants and composition, whereby the calculated CTE can deviate up to 30%^17^. The Debye-Grüneisen method models the acoustic mode of the phonons below Θ and the increased amplitudes of anharmonic vibrations at higher temperatures by the Grüneisen constant as long as structural changes such as relaxation or the approach of the melting point do not occur^13^. Deviations of the experimentally obtained CTEs from the predicted CTEs indicate regions with and without structural relaxation. For Pd_62.1_Al_27.3_Y_4.2_Ni_6.4_ the experimental CTE begins to decrease at T_r_, marking the onset of structural relaxation. Therefore, we propose the Debye-Grüneisen model as a reliable *ab initio* based method to predict CTEs of metallic glasses, provided a realistic structural model is applied, which will be discussed in the next section.

The applicability of the Debye-Grüneisen model can be justified by the work of Safarik and Schwarz^44^. The signature of a metallic glass in terms of lattice vibrations are low frequency lattice vibrations that can be modelled by Einstein oscillators. These vibrations that deviate from the Debye model are highly anharmonic. However, the anharmonic modes do not influence the bulk modulus^44^. As the bulk modulus is one major input parameter for the Debye-Grüneisen model and is not influenced by an anomalous large density of low frequency vibrations in glasses, this model can be applied for metallic glasses without the addition of Einstein oscillators in the Debye-Grüneisen model.

### *Ab initio* structural model vs. experiment

The CTE predicted by the Debye-Grüneisen model depends entirely on the atomic configuration obtained in the *ab initio* calculations. Hence, we will compare the simulated configuration with the experimentally observed one. Besides topology, glass transition temperature is a representative gauge to judge the validity of an atomic configuration. The glass transition temperature T_g_ is estimated by an empirical model introduced by Wang *et al*.^45^. They report the ratio of 1000T_g_ and MΘ_D_^2^ (the Lindemann criterion) is approximately constant for metallic glasses, considering Zr-based, Pd-based, Nd-based, La-based and Cu-based metallic glass systems, where M is the average atomic mass^45^. Based on this model, T_g_ of the calculated structure is 981 K compared to 775 K in the experiment (Fig. 1). This difference of 20% between theory and experiment is comparable with the deviations expected for the predicted CTE and even smaller compared to deviations of T_g_ calculated by classical molecular dynamics simulations for Ni-P^45,46^ and Zr-Al-Ni^47,48^ to the corresponding experimental values. Causes for this deviation might be the infinite quenching rate that increases T_g_^46^ and the small difference in composition caused by the discrete number of atoms in the supercell. Thus, the accuracy of our structural model with respect to T_g_ is within the temperature range expected from our model and literature.

**Figure 2:**
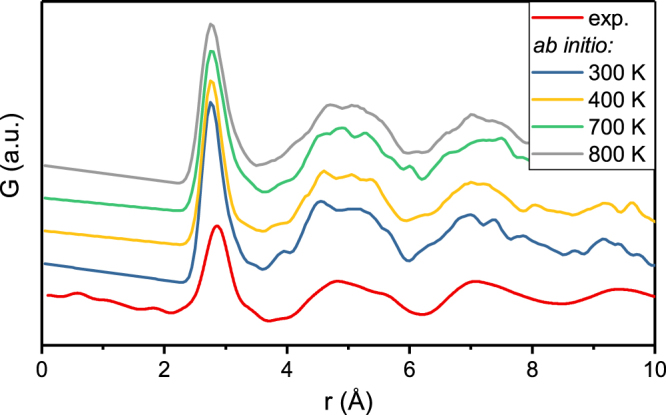
Experimental and calculated pair distribution functions of Pd_62.1_Al_27.3_Y_4.2_Ni_6.4_ at 300, 400, 700 and 800 K.

Short and medium range order have been reported in literature to have different contributions to thermal expansion^40^. Hence, we compare the calculated and experimentally obtained PDF in Fig. 2. The PDFs calculated for 300, 400, 700 and 800 K, i.e. for whole temperature range encompassing T_r_ as well as T_g_, resemble the first peak of the experimental PDF at 2.8 Å with a deviation of 0.1 Å. The second coordination shells of the calculations overlap with the experimental one. Similar to the experimental PDF, the calculated PDF for 300 K exhibits a distinct peak splitting with a peak at 4.8 Å and a shoulder at 5.3 Å, i.e. at the same positions as in the experimental case. The peak splitting is observed up to 800 K. In the third coordination shell, the calculated PDF at 300 K shows two distinct peaks which are different from the experimentally observed peak. However, the calculated third coordination shell covers the relevant r-range between 6.0 and 8.5 Å and exhibits the same asymmetric shape as the experimentally observed third coordination shell. For higher temperatures of 700 and 800 K the major peaks in the third coordination shell of the calculated PDFs are within the range of 7.5 and 7.0 Å. Despite computational constraints, the description of the short-range order up to the second coordination shell is feasible at room temperature, as the calculated PDF at 300 K resembles the experimental PDF despite the noise due to the low number of atoms. Therefore, the *ab initio* model introduced in ref.^8^ for a metalloid containing metallic glass produces a realistic atomic structure of a metalloid free metallic glass if the size of the supercell is adjusted to comprise higher coordination shells. Therefore, the increased size of the supercell compared to ref.^8^ is necessary. However, to account for structural relaxation an even larger supercell would be required. The number of 389 atoms used here, which corresponds to an increase of 338% in reference to ref.^8^, is still 1.5 times smaller than the number of atoms found in regions of correlative motion during structural relaxation in Pd-based metallic glasses^49^. To summarize the comparison of *ab initio* model and experimentally observed structure, the calculated PDF, glass transition temperature and CTE are consistent with the experimental values. Hence, the *ab initio* model represents an atomic configuration comparable to the atomic configuration obtained with magnetron sputtering.

### Structural rearrangements

**Figure 3:**
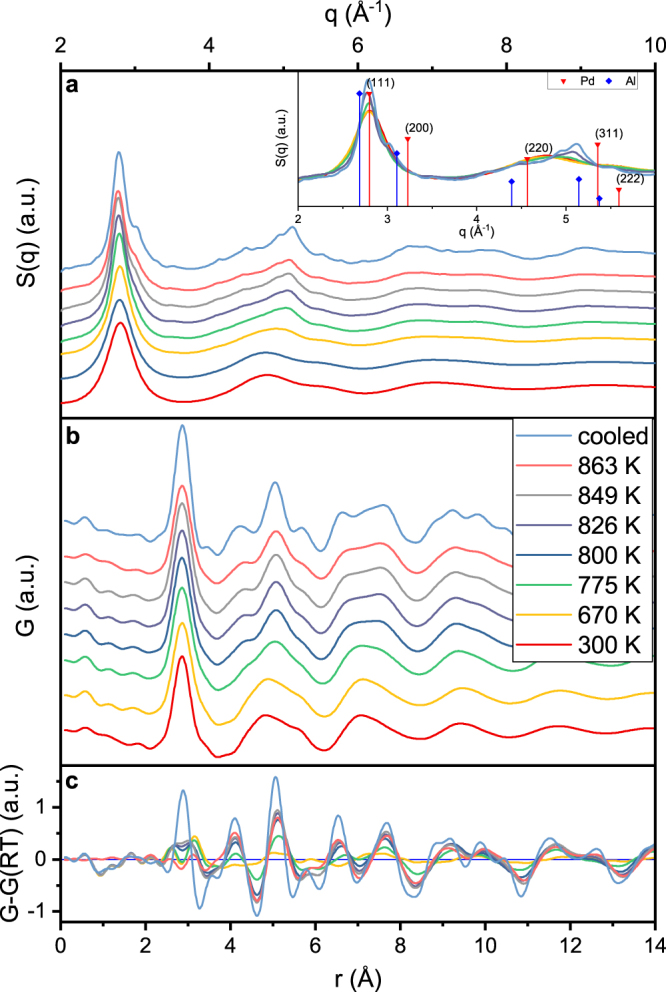
Changes in the structure of Pd_62.1_Al_27.3_Y_4.2_Ni_6.4_ during *in situ* heating and cooling. Structure factor S(Q) (**a**) and PDF (**b**) and difference between PDF at elevated temperature and room temperature (**c**) of Pd_62.1_Al_27.3_Y_4.2_Ni_6.4_ for room temperature (300 K), 670 K, glass transition temperature (775 K), the supercooled region (800, 826 and 849 K), maximum heating temperature and the cooled down sample. The inset in (**a**) shows the peak positions of the Bragg peaks of the fcc-phases of the main constituents Pd and Al.

**Figure 4:**
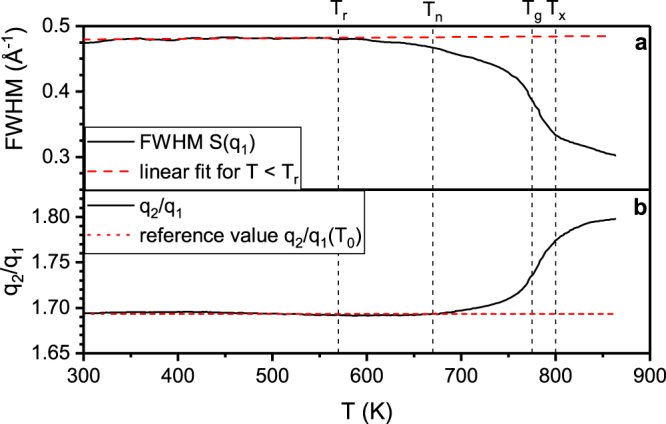
Changes in the structure factor with temperature. (**a**) Full width half maximum (FWHM) of principal peak of the structure factor as a function of temperature in the heating section, the dashed line is a linear fit of the FWHM below T_r_ (**b**) second to first peak ratio q_2_/q_1_ of the structure factor as a function of temperature in the heating section, the dashed line marks a constant q_2_/q_1_ ratio.

The deviation of (Q(T_0_)/Q(T))^3^ from a linear dependence on T and the deviation of the CTE from the theoretical prediction at T_r_ indicate the onset of structural rearrangements, which have implications on mechanical properties^43^. Temperature induced mechanical property and phase stability changes are of key importance for structural applications^41^. Thus, to investigate the atomic rearrangements, we employed a refined structural analysis. To understand the influence of structural rearrangements on the medium range order, we compare the structure factor obtained by high energy X-ray diffraction at room temperature; T_n_ = 670 K, at which the CTE changes from positive to negative values; T_g_ = 775 K; and the maximum heater temperature of 863 K (Fig. 3(a)). From the comparison of the structure factor at RT and 670 K we observe a slight peak shift towards lower wavevectors, while the peak width begins to decrease at T_r_ (Fig. 4(a)). This indicates an onset of structural rearrangements towards a more ordered structure, which is consistent with the comparison of predicted and measured CTE. Between 670 K and T_g_ = 775 K, the second peak of S(Q) (Fig. 3(a)) shifts significantly towards larger q values from 4.77 to 4.90 Å^−1^. Furthermore, the ratio of first and second peak positions q_2_/q_1_ (Fig. 4(b)) deviates from a constant value above 670 K. Hence, starting at T_n_ = 670 K inelastic structural changes occur that cannot solely be attributed to thermal expansion^33^. The changes in S(Q) (Fig. 3(a)) indicate that atomic rearrangements become more pronounced in the supercooled liquid region between T_g_ and 863 K. In the supercooled liquid region, the principal peak develops a shoulder at 3 Å^−1^ and decreases in height due to atomic rearrangements, while a peak sharpening of the main peak in the second coordination shell is observed, indicating the onset of the Bragg peaks and hence structural rearrangements towards the formation of a crystalline phase. These small Bragg peaks are irreversible and clearly visible in the cooled down sample, which consists of nanocrystals in an amorphous matrix after the heating cycle.

Comparing the peak positions of S(Q) to the Bragg peaks of the crystalline phases of the main constituents Pd and Al (inset in Fig. 3(a)), the principle peak shifts towards the (111) peak of Pd, while the shoulder of the principal peak is near the Bragg peaks of the (200) plane. The main peak of the second coordination shell matches the (311) peak of Al, while its shoulders are between the (220) and (222) peaks, respectively, of both elements. Thus, the fcc crystalline structures appear to evolve via fcc-like short-range ordered clusters before the onset of crystallisation. Similar observations have been reported by Voyles *et al*. for the amorphous to crystalline transition of Si^50^.

Despite the nanocrystalline phase in the glass present in the cooling region, the CTE for the glass and a nanocrystal-glass composite material are equal within the error of the measurement. This equivalence of CTEs may be explained by the portion of the amorphous phase and the similarity of the CTE of the major constituent Pd. The rather broad principle peak of S(Q) of the cooled down sample in Fig. 3(a) and the small Bragg peak indicate a considerably large amorphous portion after the heating and cooling cycle. Additionally, the CTE of 3.4 × 10^−5^ K^−1^ for main constituent Pd^13^ is in agreement with the calculated CTE for the amorphous phase. Thus, the difference of CTE between crystalline and amorphous phase might be small, wherefore the CTE of the nanocrystal-glass composite is equal to the amorphous CTE.

Complementary to the medium-range order analysis, the development of short-range order is probed by employing real space PDFs. The experimentally obtained real space reduced PDF G(r) shown in Fig. 3(b) underlines the structural changes during heating. Corrugations at bond distances lower than 2.1 Å are artefacts from the Fourier transform of S(Q) to G(r)^51^. The first peaks of G(r) are at distances of 2.9, 4.8, 7.1 and 9.4 Å. The first peak indicates Pd-dominated bonding, as its position is at the distance of Pd-Pd bonds in icosahedral coordination. The G(r) at RT and 670 K resemble each other in terms of peak shape and position. Within the temperature range of RT to 863 K the shape of the nearest neighbour shell remains unchanged. However, the peak height is decreasing until T_g_ by 13%, while the full width half maximum (FWHM) is increasing until T_g_ by 8%. The difference between the PDF at elevated temperatures and PDF at 300 K (Fig. 4(c)) shows an increase of the peak flanks with a larger portion at the right flank, while the height at the peak centre decreases. Thus, thermal rejuvenation might occur, that broadens the atomic bond length distribution in the first coordination shell and thereby increases the disordering^52^ by thermally activated atomic movements. However, the thermal rejuvenation of the short range order does not affect the average atomic volume, as the FWHM of the principle peak of S(Q) begins to narrow and the (Q(T_0_)/Q(T))^3^ ratio begins to change already at 570 K. Since the average volume is represented by the principle peak of S(Q), the changing (Q(T_0_)/Q(T))^3^ ratio indicates free volume annihilation^31^ concurrent to thermal rejuvenation of the short range order. As can be derived by the continuously changing slope of Q(T_0_)/Q(T))^3^ towards a more negative value, the free volume annihilation rate in the medium range order increases up to T_g_, where the average atomic volume begins to increase again. Thermal rejuvenation stops at the glass transition temperature, where structural relaxation sets on as the FWHM decreases and principle peak height increases^53^ strongly up to T_x_, where the slope decreases and thus structural relaxation slows down. During structural relaxation, the right flank of the first coordination shell decreases (Fig. 4(c)), while the left flank is increased compared to the reference. Thus above T_g_, free volume annihilation takes place in the short range order due to removal of large atomic bond distances^53^. This structural relaxation towards a more ordered structure at T_x_ is consistent with the continuous development of a more ordered (crystalline) structure, which has been already observed by the evolution of the Bragg peaks in S(Q).

While free volume is annihilated between the nearest neighbours above T_g_, peak splitting is observed for the second to fifth coordination shell, indicating chemical rearrangement. Whereas the major subpeak of the second coordination shell below T_g_ is at 4.8 Å, the major peak at T_g_ is at 5.1 Å with shoulders at 4.3 and 5.6 Å. These three subpeaks become more pronounced with increasing temperature in the supercooled region. Also in the third coordination shell the subpeak at 7.4 Å, which was not dominating below T_g_, evolves at T_g_ and eventually dominates the third coordination shell in the supercooled region. Shoulders evolve also in the fourth coordination shell in the supercooled region. This evolution of subpeaks in the second to fourth coordination shell hints to a rearrangement of local chemical environment^53^ of Pd, which is the major constituent in this metallic glass.

**Figure 5:**
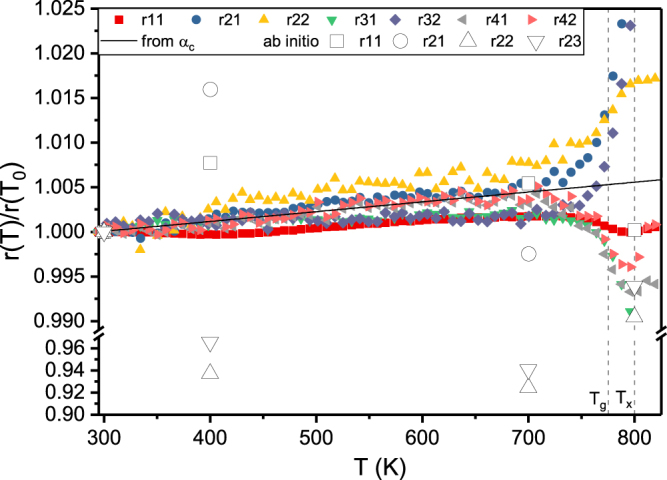
Relative shift of peak positions r_ij_ of G(r) as a function of temperature of the j^th^ peak in the i^th^ coordination shell. The solid line marks the relative peak shift expected from the average CTE α_c_ obtained from the structure factor S(Q). Empty symbols represent relative peak shifts of the calculated PDF from Fig. 2. T_g_ and T_x_ are the glass transition and crystallisation temperature, respectively.

The different behaviour of short and medium range order in terms of free volume annihilation, thermal rejuvenation and relaxation hints to a heterogeneous atomic environment on the short range order level. To understand the relation of the individual coordination shells in the short range ordered region and the thermal expansion, we calculated the temperature induced relative shift of peak positions of the individual coordination shells. Figure 5 depicts the relative shift of the peak positions r_ij_ of the PDF (indices indicating the j^th^ peak in the i^th^ coordination shell) during heating. The average thermal expansion obtained from the shift of the principle peak of S(Q) is indicated by a solid line and supposed to be approximately the macroscopic thermal expansion^40^. The reference peak positons at T_0_ = RT for r_11_, r_21_, r_22_, r_31_, r_32_, r_41_ and r_42_ are 2.86, 4.75, 5.05, 6.95, 7.29 9.26 and 9.54 Å, respectively. From Fig. 5 we learn that the thermal expansion of the coordination shells is inhomogeneous. The first and third coordination shell contract compared to the macroscopic thermal expansion, while the second coordination shell expands more than by the macroscopic thermal expansion. The expansion of the fourth coordination shell matches the macroscopic thermal expansion. The peak shifts of the first to third coordination shells are due to rearrangements on the atomic level (Fig. 3(b,c)). Comparing the cooled down sample to the initial sample, the second peak at 3.4 Å forms in the first coordination shell, might be associated with the bonds involving Y, while the second coordination shell consists of three distinct peaks at 4.2, 5.0 and 5.7 Å instead of one peak containing two subpeaks. The difference of the PDF of the cooled down sample and the pristine sample (Fig. 3(c)) reveals a sharpening of the peak of the first coordination shell, indicating a narrower bond length distribution. The distinct peaks in the second coordination shell verify the increased ordering in the sample after the heating cycle. However, in Fig. 5 we observe that the higher coordination shells reflect the macroscopic thermal expansion obtained from the principal peak shift in S(Q)^40^. This can readily be understood based on the inverse relationship between wavenumber and bond distance. The principal peak of S(Q) contains the major information of the medium range order^34,54^. Thus, it is learned from the analysis of both S(Q) and G(r) that the macroscopic thermal expansion is reflected by the medium range order, while irreversible changes take place in the heterogeneous atomic environment of the short and near medium range order up to bond distances of 13.2 Å, which is in agreement to literature reports^40^ on CuZr metallic glasses. During structural relaxation, the exchange of atoms with different sizes between the heterogeneous coordination shells is possible^55^, allowing the contraction and expansion of coordination shells as is the case for the first to third coordination shell, leading to inhomogeneous thermal expansion on the atomic scale. Following the shifts of the peak positions in Fig. 5 we observe a significant change in slope around 745 K, inferring an increased rate of structural rearrangements. The inflection point at 775 K corresponds to the glass transition temperature T_g_ observed in (Q(T_0_)/Q(T))^3^. Further heating leads to a minimum at 800 K. This change of slope in real space is identified as crystallisation temperature T_x_^34^. This temperature coincides with the flattening of the width of the principle peak of the structure factor and of the q_2_/q_1_-ratio in Fig. 4. The onset of crystallisation at T_x_ is consistent with the observation of Bragg peaks in the structure factor (Fig. 3(a)) inferring nanocrystal formation within a glassy matrix.

The empty symbols in Fig. 5 show the relative peak shifts of the calculated PDFs in Fig. 2 and underline the lack of structural relaxation in the *ab initio* model. The peak positions of the calculated PDF do not shift in accordance with the experiments, but show a strong scattering. The scattering is likely caused by the small volume of the supercell, which introduces noise into the calculated PDF that is also observed for bond distance larger 4 Å (Fig. 2). Hence, a direct determination of CTEs from the shift of peak positions in real space is not feasible. However, as shown in Fig. 2 the description of the short-range order up to the third coordination shell by *ab initio* calculations is possible at room temperature. This enables the prediction of CTEs with the Debye-Grüneisen model, which is more efficient in terms of time and resources compared to experimental methods.

## Conclusion

The average room temperature CTE of the Pd_54.7_Al_23.5_Y_7.8_Ni_11.3_ thin film metallic glass obtained from Debye-Grüneisen model is 3.4 × 10^−5^ K^−1^, while the experimental CTEs calculated from *in situ* heating and cooling cycles in synchrotron X-ray diffraction experiments are 3.5 ± 0.3 × 10^−5^ and 3.4 ± 0.1 × 10^−5^ K^−1^, respectively. Hence, we observe excellent agreement between the experimentally and theoretically obtained CTEs. Therefore, we propose the Debye-Grüneisen model as a reliable *ab initio* based method for predicting the CTE of metallic glasses. A comparison between the calculated and experimental PDF shows that the experimentally observed structure at room temperature is well resembled by the *ab initio* model. The temperature induced structural relaxation is not covered by our model containing 389 atoms, which is the cause for the deviation between theoretical and experimental CTE data for temperatures above T_r_. The deviation originates in the rearrangement of atoms and the annihilation of free volume during structural relaxation within the experiment.

The *in situ* high energy X-ray diffraction structure analysis reveals an inhomogeneous expansion of the individual coordination shells. For the Pd_62.1_Al_27.3_Y_4.2_Ni_6.4_ thin film metallic glass the macroscopic thermal expansion is dominated by the medium range order, while thermal rejuvenation and atomic rearrangements are observed in the short range order. Structural relaxation of the average structure starts at 570 K. The glass transition temperature of Pd_62.1_Al_27.3_Y_4.2_Ni_6.4_ is 775 K, which we identified through a change of signs of the CTE. Above T_g_, the peaks of S(Q) shift towards the positions of the Bragg peaks of the crystalline phases of the main constituents. Hence, before the onset of crystallisation a continuous evolution towards a fcc structure via a fcc-like arrangement of short-range ordered clusters is proposed, leading to the formation of a nanocrystal-glass composite material. The crystallisation temperature of 800 K is 25 K higher than T_g_. The akin CTEs of the metallic glass and nanocrystal-glass composite material suggest thermal expansion based on the same mechanism of coordinated vibrations of strings of atoms next to highly defective areas in Pd-based metallic glasses and nanocrystal-glass composites.

Based on the average atomic volume, we observe free volume annihilation in the medium range order that exceeds the volume expansion at 670 K leading to a negative thermal expansion. Above T_g_, free volume annihilation takes place in the short range order, while the average atomic volume increases in the supercooled region.

### Data availability statement

The data generated and analysed during this study are available from the corresponding author on reasonable request.
